# Pedigree analysis of atrial fibrillation in Irish wolfhounds supports a high heritability with a dominant mode of inheritance

**DOI:** 10.1186/s40575-019-0079-y

**Published:** 2019-12-09

**Authors:** Samantha L. Fousse, William D. Tyrrell, Mariellen E. Dentino, Frances L. Abrams, Steven L. Rosenthal, Joshua A. Stern

**Affiliations:** 1 Department of Medicine and Epidemiology, University of California Davis - School of Veterinary Medicine, Davis, CA USA; 2 CVCA: Cardiac Care for Pets, Leesburg, VA USA; 3 Irish Wolfhound Foundation, Lititz, PA USA; 4 CVCA: Cardiac Care for Pets, Towson, MD USA

**Keywords:** Veterinary, Genetics, Arrhythmia, Breeding, Dog, Canine

## Abstract

**Background:**

Atrial fibrillation (AF) is the most common arrhythmia in dogs. The Irish Wolfhound breed has a high prevalence of AF making them an ideal breed to investigate possible genetic contributions to this disease. The aim of this study was to perform a heritability analysis in North American Irish Wolfhounds using phenotype data from cardiac screenings performed between 2000 and 2019 in order to determine how much of this disease can be attributed to genetics compared to environmental causes. The second aim was to determine the disease mode of inheritance to help inform prevention and breeding practices.

**Results:**

There were 327 Irish Wolfhounds diagnosed with AF and 136 Irish Wolfhounds over 8 years of age without AF. The estimated mean (95% confidence interval) heritability of AF in Irish Wolfhounds was 0.69 (0.50–0.86). The pedigree was consistent with a dominant mode of inheritance.

**Conclusion:**

Results of this study indicate a strong genetic contribution to AF in Irish Wolfhounds and suggest that future research to identify causative genetic mutations is warranted.

## Plain English summary

Atrial fibrillation (AF) is a common heart rhythm disorder in the dog that may result in clinical signs such as fainting or heart failure. Certain dog breeds, such as the Irish Wolfhound, get AF more often compared to other dog breeds. In humans, AF has high heritability, meaning that if an individual has a relative with AF, they are more likely to get AF due to genetics. This study aimed to determine if genetics contributes to AF in Irish Wolfhounds by looking at the family history of dogs with and without AF from 2000 to 2019. This study determined that in Irish Wolfhounds, AF has a high heritability. Pedigree analysis was consistent with an autosomal dominant mode of inheritance in the Irish Wolfhound meaning that a dog with AF needs to have a parent with AF as well. However, autosomal recessive and polygenic modes of inheritance cannot be definitively excluded. This has important implications for Irish Wolfhound breeding and suggests that additional research to figure out the genetic cause of AF in Irish Wolfhounds is necessary.

## Background

Atrial fibrillation (AF) is the most common arrhythmia in dogs [1]. It is characterized by an “irregularly irregular” R-R interval with the absence of identifiable P waves on an electrocardiogram. The irregular frequency of ventricular contraction is due to the disorganized electrical activity of the atria [2]. This may result in reduced cardiac output leading to clinical signs such as exercise intolerance, fainting, and congestive heart failure [3].

Atrial fibrillation has a prevalence of 0.10–0.43% in dogs seen at a veterinary clinic [4–6]. When adjusting the prevalence for dogs with clinical heart disease, the prevalence of AF rises to 6.27–10.5% [4, 5]. Certain dog breeds such as the Irish Wolfhound have a high prevalence of AF [7–9]. Specifically in North America, the prevalence of AF in Irish Wolfhounds ranges from 8.9–12% overall [7, 10]. AF is typically an age-associated disease; if you adjust the prevalence to only include dogs ≥8 years it increases to 28% [7]. However, this disease can still occur in younger dogs often with more severe clinical signs [8].

Atrial fibrillation is a complex disease that is influenced by both genetics and the environment [11, 12]. In humans, AF is highly heritable [13]. For example, an individual’s risk of developing AF increases if their parent or first degree relative has AF [14, 15]. Despite AF being described in several veterinary species [16–19], the only species with a reported heritability for AF is the Standardbred racehorse [20].

Atrial fibrillation has been associated with dilated cardiomyopathy (DCM) in Irish Wolfhounds in several studies [8, 10, 21–23]. In humans, AF and DCM can be due to genetically distinct causes or caused by the same mutation [12, 24, 25]. The same may be true for Irish Wolfhounds because within the breed each disease can occur simultaneously, precede the diagnosis of the other, or occur in isolation [8, 22, 23, 26]. Another possibility is that some diagnoses of DCM may be tachycardia-induced cardiomyopathy due to the increased heart rate from the arrhythmia [27]. Irish Wolfhound hearts with AF, DCM, or both cannot be differentiated based on histopathology suggesting a link between the two diseases [28]. Although AF and DCM may be linked, many of the genetic studies have thus far focused on DCM in Irish Wolfhounds or have combined AF and DCM dogs into a single group instead of examining AF dogs in isolation [23, 29, 30]. Fortunately, The Irish Wolfhound Foundation has been gathering annual electrocardiograms on a population of Irish Wolfhounds in North America since 2000 [7]. This large dataset along with Irish Wolfhounds being overrepresented compared to other dog breeds in the prevalence of AF, is ideal to investigate the contribution of genetics to AF. Therefore, the aim of this study was to perform a heritability analysis in North American Irish Wolfhounds to determine the genetic contribution to AF in the Irish Wolfhound breed. A second aim was to determine the mode of inheritance for AF in Irish Wolfhounds.

## Results

There were 327 (153 males, 174 females) Irish Wolfhounds diagnosed with AF. There were 40 affected Irish Wolfhounds that had siblings diagnosed with AF. Specifically, 5 sets of four, 5 sets of three, and 30 sibling pairs with AF. There was not a statistically significant difference between the number of males and females diagnosed with AF (*p* = 0.25). There were 136 (50 males, 86 females) Irish Wolfhounds over 8 years of age that were never diagnosed with AF. There were significantly more females in the control group compared to males (*p* = 0.002).

The estimated mean heritability with a 95% confidence interval for AF in Irish Wolfhounds was 0.69 (0.50–0.86), while the median heritability was 0.70. There were 12 individuals where the phenotypes of both parents were known. There were 95 individuals where the phenotype of at least one parent was known. There were 356 individuals where the phenotype of either parent was unknown. Of the 561 parents of affected Irish Wolfhounds with unknown phenotype status: 9 were not old enough to meet the age restriction of 8 years at the time of writing, 93 died before the age restriction of 8 years, 21 had an arrhythmia that was not AF which disqualified them as a control, and the remainder had unknown cardiac status.

There were 74/327 (22.6%) AF individuals that had at least one parent with AF. There were 13/136 (9.6%) control Irish Wolfhounds that had at least one parent with AF. There were 27 instances of an affected male transmitting AF to a son. No dam had over five AF-affected offspring. There were four sires with over five AF-affected offspring each; two sires had AF and two sires had unknown heart status. The distribution of parental phenotypes for cases and controls is available in Table 1.

**Table 1:** Distribution of parent phenotypes for Irish Wolfhounds with atrial fibrillation or without atrial fibrillation

Sire Phenotype	Dam Phenotype	Case	Control	Total
Atrial fibrillation	Atrial fibrillation	3	1	4
Atrial fibrillation	Unknown	46	3	49
Unknown	Atrial fibrillation	19	7	26
Atrial fibrillation	Normal	5	1	6
Normal	Atrial fibrillation	1	1	2
Unknown	Normal	8	7	15
Normal	Unknown	2	3	5
Normal	Normal	0	0	0
Unknown	Unknown	243	113	356
Total		327	136	463

**Fig. 1:**
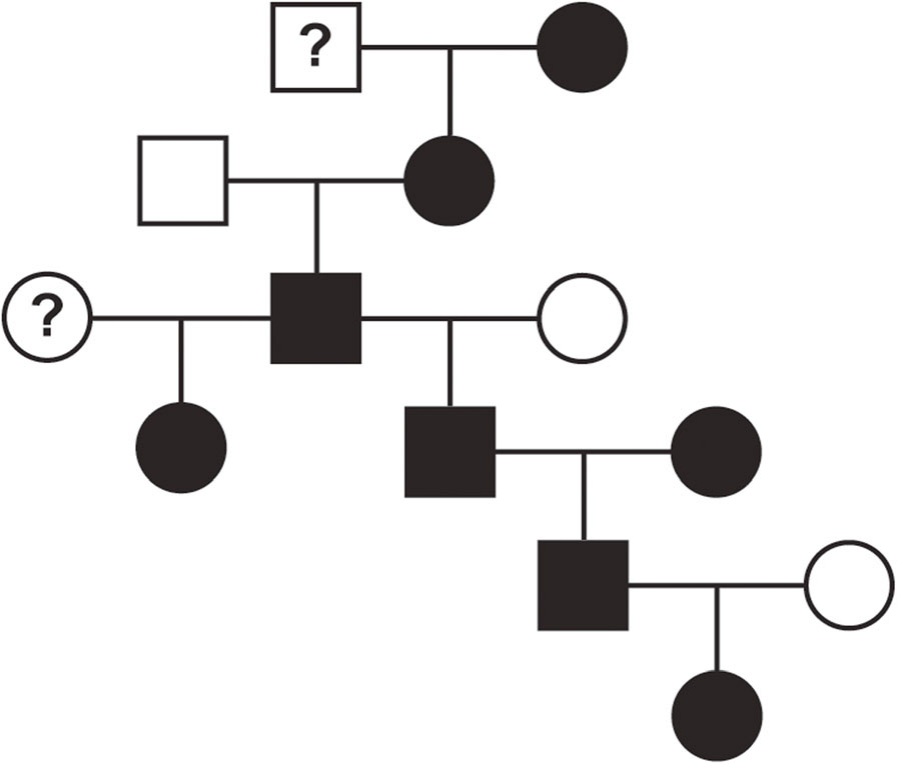
An Irish Wolfhound family pedigree that supports a dominant mode of inheritance for atrial fibrillation. Square = male. Circle = female. White = no atrial fibrillation over eight years of age. Black = diagnosed with atrial fibrillation at any age. Question mark = no cardiac phenotype information available to classify as affected or unaffected

A subset of the overall pedigree is displayed in Fig. 1. Figure 1 contains a family of 13 Irish Wolfhounds (8 females, 5 males) spanning 6 generations. An affected individual is identified in every generation. There were 5 affected females and 3 affected males. Note that affected males are siring both affected male and female offspring. There are 4 matings for which both parents are known: three affected/unaffected and one affected/affected combination. There were no unaffected-to-unaffected matings in the pedigree.

X-linked recessive inheritance can be ruled out because there was no statistically significant difference between the number of males and females diagnosed with AF. X-linked dominant inheritance can be ruled out because an affected male transmitted the disease to a son 27 times in the pedigree. A dominant mode of inheritance is supported in a familial cluster because an affected individual is present in every generation when phenotype information is available (Fig. 1). Additionally, of 84 cases with at least one parental phenotype known, 74 (88.1%) have at least one parent diagnosed with AF and no case had two normal parents.

## Discussion

This is the first study to characterize the genetic contribution of AF in Irish Wolfhounds. The heritability estimate of AF for Irish Wolfhounds is 69%. This is similar to what was found in a human Danish twin study [13], but higher than what was found in Standardbred horses [20]. Additionally, this is higher than the previously reported heritability of dilated cardiomyopathy in Irish Wolfhounds, another common cardiac disease in the breed that is frequently associated with AF [30]. The high heritability estimate obtained in Irish Wolfhounds suggests that genetic effects play an important role in disease expression. This may allow the disease to be carefully selected against to reduce its incidence in the breed.

Findings are consistent with a dominant mode of inheritance for AF in Irish Wolfhounds. This dominant mode of inheritance is similar to what has been found in some human familial forms of AF [31], but AF in humans is complex with many genes contributing to the development of the disease. The Irish Wolfhound pedigree is suggestive of autosomal dominant because the majority of cases (88.1%) with a known parental phenotype have at least one parent diagnosed with AF. Although based on these numbers it seems unlikely, an autosomal recessive or polygenic mode of inheritance cannot be definitively ruled out as many of the parental phenotypes of the cases and controls were unknown.

Irish Wolfhound DCM is inherited as a major gene model with additional polygenic and sex dependent components [30]. Although the polygenicity of AF cannot be ruled out in this study, X-linked inheritance was ruled out. The polygenicity of DCM in Irish Wolfhounds is supported by a genome-wide association study identifying multiple loci [29] and a study that suggests multiple loci better predicts DCM onset compared to a single loci [23]. To the authors’ knowledge, no loci have been identified in dogs with AF as the sole criteria for a case. Future genomewide association studies using AF cases compared to controls would be useful to confirm whether AF is polygenic or due to a single gene, as well as determine if AF and DCM share the same genetic loci.

Although a dominant mode of inheritance permits rapid selection against a disease, caution is advised as this approach can dramatically limit genetic diversity, particularly when the disease prevalence is high (as seen with AF in Irish Wolfhounds). Ideally, this insight into heritability and pattern of inheritance will lead to continued research and discovery of a genetic mutation associated with AF in the Irish Wolfhound breed. To avoid decreasing genetic diversity in the breed, a genetic test would be useful to permit a slower reduction in the incidence of disease by avoiding breeding homozygous-mutant dogs or breeding two heterozygous individuals. A genetic test would also allow the identification of individuals who may develop AF after breeding age or need to be screened more often for AF. Another way to avoid substantially decreasing genetic diversity is by limiting removal of individuals from the breeding pool to only those that are diagnosed at a young age or have an aggressive form of AF that is associated with Irish Wolfhound type cardiomyopathy [7].

A limitation of this study is that many of the cases and controls did not have parental phenotypes. This may represent a bias in the population due to the way data was collected. The screening events occurred at dog shows with the recommendation that relatives of AF individuals receive follow-up by a local veterinary facility and veterinary cardiologist. Although efforts were made to encourage dogs diagnosed with AF to come to screenings, if a dog is diagnosed with AF by a local veterinarian, those individuals may never attend the dog show screening events and thus be underrepresented in this study.

The lower number of controls compared to cases in the study is likely due to the difficulty of obtaining electrocardiograms on older Irish Wolfhounds. Lowering the age cutoff would allow more parental and litter phenotypes to be known but would also reduce the confidence that the dog would not have developed AF in the future. The median lifespan of an Irish Wolfhound is 7.5 years [32], however 8 years old was the age used as the control cutoff because a diagnosis of AF is known to increase with age and the investigators considered phenotypic certainty for the unaffected group to be of paramount importance for this study [7]. Consequently, many dogs passed away before they could be considered a control. Although the Irish Wolfhound Foundation subsidized the screening costs for veteran dogs, senior Irish Wolfhounds are beyond typical breeding age, therefore fewer individuals are likely to receive a cardiac screening unless warranted due to clinical signs.

Another limitation is that only a diagnosis of AF was used for phenotyping and not a diagnosis of AF and DCM. To reduce limiting the number of phenotyped cases and controls, only AF was included in phenotyping for this study because the majority of dogs in the historical dataset did not receive an echocardiographic examination. Only including AF-affected dogs would prevent exclusion of affected individuals that died before developing DCM or those that never received a follow-up heart test. Unfortunately, this means that controls that had DCM but not AF would not have been excluded or cases that only had DCM would not be included.

Many heritability studies compare inbreeding coefficients between cases and controls. However, pedigree-based inbreeding coefficients were not calculated in this study because a previous study found that due to recent population expansion in the Irish Wolfhound breed, the 10-generation inbreeding coefficient is underestimated and inaccurate [33].

## Conclusions

Irish Wolfhounds have a high heritability estimate for atrial fibrillation. A pedigree analysis suggests a dominant mode of inheritance. Future research is necessary to identify the genetic variant(s) driving these high heritability estimates. Once identified, the development of a genetic test is recommended to assist breeding efforts aimed at reducing the incidence of AF in the breed.

## Methods

Historical data was obtained from the Irish Wolfhound Foundation. The Irish Wolfhound Foundation collected this data with owners’ consent as part of cardiac screening efforts held primarily at dog show events between the years 2000–2019. Case inclusion criteria was a diagnosis of AF at any age obtained by 6-lead electrocardiograms recorded in conscious, non-sedated, standing Irish Wolfhounds evaluated by a board-certified veterinary cardiologist. Control inclusion criteria was any Irish Wolfhound over 8 years of age that had no identified arrhythmias by 6-lead electrocardiograms recorded in conscious, non-sedated, standing Irish Wolfhounds evaluated by a board-certified veterinary cardiologist. Case and control demographics were recorded and a chi-square test was used to determine if sex had any influence on disease classification.

Dogs were included in the pedigree analysis portion of this study if at least 3 generations of pedigree data was available. Pedigree data was obtained from a free and publicly available database for Irish Wolfhounds [34]. The pedigree was manually generated using Adobe Illustrator. A proposed mode of inheritance was identified using previously established definitions of autosomal, x-linked, recessive and dominant [35, 36].

### Heritability analysis

A pedigree containing three generations for each case and each control was used to calculate heritability using MCMCglmm Package in R-software [37]. The heritability analysis was performed using a binary category analysis (i.e. control or affected with AF). This assumes a threshold model for the liability to disease meaning that the underlying, unobservable risk for AF is assumed to be continuous. However, a diagnosis of AF only occurs when the risk exceeds a threshold of Tao = 0. A generalized mixed model with probit link function was used. A correction for ascertainment bias is not necessary because the dogs were diagnosed as either case or control as part of a breed-wide, annual, and recommended screenings instead of centering around a proband.

## Data Availability

The datasets used and/or analyzed during the current study are available from the corresponding author on reasonable request.

## Abbreviations

AF: Atrial fibrillation
DCM: Dilated cardiomyopathy
